# FIRST: Enhancing Agency Dementia Capability through Dementia-Specific Interventions and Supportive Services

**DOI:** 10.1093/geroni/igab046.2761

**Published:** 2021-12-17

**Authors:** Elizabeth Wellbrock, Joanna Hutchinson

**Affiliations:** County of San Diego Health and Human Services Agency, San Diego, California, United States

## Abstract

Through the First Identify and Refer then Serve and Track (FIRST) Project, individuals and caregivers have critical connections to community organizations and resources to learn ways to handle living with memory problems. The FIRST project integrated new practices into existing programs to address gaps in service and piloted a new dementia-specific case management program. The initial intervention is a system-level change within the County of San Diego’s Aging & Independence Services (AIS) department to identify, pilot, and implement a brief Alzheimer’s Disease and Related Dementias (ADRD) screening tool. The tool was used by non-clinical personnel to identify potential ADRD cases. Individuals who screened positive for possible ADRD were referred to their physicians for an accurate diagnosis. The second intervention consisted of two components: a behavioral symptom management intervention for social workers to use in the home with caregivers and a dementia-specific case management program (including respite care) to improve quality of life and future planning for those with ADRD living alone or with a family caregiver. As of January 2021, 536 clients across several AIS programs have been screened for ADRD, of which, 60% screened positive. FIRST case management has served 196 clients, 70 who lived alone and 126 who lived with their caregiver. Respite was provided to 98 clients totaling to 3,666 hours. This poster evaluates the effectiveness of the program components in increasing dementia capability of an agency, and where applicable, its effect on caregiver burden and self-efficacy.

